# Multidimensional Transition Metal Complexes Based on 3-Amino-1*H*-1,2,4-triazole-5-carboxylic Acid: From Discrete Mononuclear Complexes to Layered Materials

**DOI:** 10.3390/molecules200712341

**Published:** 2015-07-07

**Authors:** Bing Liu, José A. Fernandes, João P. C. Tomé, Filipe A. Almeida Paz, Luís Cunha-Silva

**Affiliations:** 1 Department of Chemistry, CICECO–Aveiro Institute of Materials, University of Aveiro, Campus Universitário de Santiago, 3810-193 Aveiro, Portugal; E-Mails: bliu_1203@163.com (B.L.); jafernandes@ua.pt (J.A.F.); 2 REQUIMTE/LAQV & Department of Chemistry and Biochemistry, Faculty of Sciences, University of Porto, 4169-007 Porto, Portugal; 3 Department of Chemistry, University of Aveiro, QOPNA, Campus Universitário de Santiago, 3810-193 Aveiro, Portugal; E-Mail: jtome@ua.pt; 4 Department of Organic and Macromolecular Chemistry, Ghent University, B-9000 Ghent, Belgium

**Keywords:** 1,2,4-triazole, crystal structures, supramolecular organization

## Abstract

The synthesis and structural characterization of five transition metal complexes with different dimensionality and incorporating residues of 3-amino-1*H*-1,2,4-triazole-5-carboxylic acid (H_2_atrc) is reported: [Zn(Hatrc)_2_(H_2_O)] (**1**), [Mn(Hatrc)_2_(H_2_O)_2_]·2H_2_O (**2**), [Fe_2_(Hatrc)_4_(OH)_2_]·6H_2_O (**3**), [Cd(Hatrc)_2_(H_2_O)]*_n_* (**4**), and [Mn(atrc)(H_2_O)]*_n_*·*n*H_2_O (**5**). These materials could be prepared from solution (**1**–**3**), diffusion (**4**), or hydrothermal reactions (**5**) with various anions and L:M ratios. Structural details were revealed by single crystal X-ray diffraction. The discrete units composing compounds **1**–**3**, the polymeric 1D chain of **4** and the 2D layer of **5** are further extended into 3D supramolecular architectures through the formation of hydrogen bonds.

## 1. Introduction

The rational strategy to design new coordination complexes, especially Metal-Organic Frameworks (MOFs) or coordination networks, by self-assembly has received remarkable interest due to their fascinating structural features and potential to be applied as novel functional materials [1,2,3,4,5,6,7]. The final assembly can be influenced by numerous factors, such as geometric requirements of metal centers, shape and nature of the ligands, reaction routes, solvents, templates, pH of the reactive medium and counterions [8,9,10,11,12]. At the moment, immense research activity is focused on integrating the advantages of metal centers and organic spacers in a complementary way. The inorganic component imparts magnetism, mechanical strength and thermal stability, while the organic part offers a way to improved luminescence, structural diversifications, and processability [13,14,15,16]. The effort exerted in this field promotes the understanding of the structure-property relationship, being attributed to the purposeful design and controlled synthesis of the aimed complexes [17,18,19,20,21].

One fruitful selection of the organic building units relies on five-membered N-heterocycles, such as pyrazole, imidazole, triazole, tetrazole, which are good representatives for small and simple organic bridging ligands. 1,2,4-Triazole residues, presenting a hybrid of pyrazole and imidazole, represent a class of ligands that can combine different substituent groups, thus constituting a good building block for the preparation of metal complexes and materials with structural, optical and magnetic properties [22,23,24,25,26,27,28,29,30,31]. Herein, 3-amino-1*H*-1,2,4-triazole-5-carboxylic acid (H_2_atrc) [32], comprising a triazole ring with three N-donors and a carboxylic acid group, was chosen as the organic building unit, being expected to lead to the formation of polynuclear compounds and multidimensional frameworks. A meticulous survey in the literature and in Cambridge Structural Database (CSD, Version 5.36 updated November 2014) [33] revealed the existence of only five structures with residues of H_2_atrc (this molecule being the sole organic ligand). Two of these known compounds comprise discrete complexes ([Cd(Hatrc)_2_(H_2_O)_2_] [34] and (NH_4_)_2_[Cd(Hatrc)_4_] [35]), whereas the other two are isotypical 3D MOFs ([M(Hatrc)_2_(H_2_O)]_n_; M^2+^ = Sr^2+^ or Ba^2+^) [36]. The remaining compound is an interesting tubular structure formed by the crystal packing of [Cu(atrc)(H_2_O)] complex units, whose channels have an internal diameter of *ca.* 8.5 Å. Clusters of 12 molecules of water of crystallization are included in the channels [37].

The rarity of (H)atrc-M systems is most likely caused by the fact that H_2_atrc produces highly insoluble precipitates, even when using mild synthetic conditions. As part of our ongoing research work dealing with the preparation of functional coordination complexes [38,39,40,41] and new MOFs [42,43,44,45,46,47], we have isolated a series of transition metal (TM) complexes with H_2_atrc anionic residues. These materials show distinct dimensionalities and were prepared by several methods: [Zn(Hatrc)_2_(H_2_O)] (**1**), [Mn(Hatrc)_2_(H_2_O)_2_]·2H_2_O (**2**) (mononuclear complexes), [Fe_2_(Hatrc)_4_(OH)_2_]·6H_2_O (**3**; dinuclear complex) were isolated by slow evaporation; [Cd(Hatrc)_2_(H_2_O)]*_n_* (**4**; 1D MOF) was prepared by diffusion method; [Mn(atrc)(H_2_O)]*_n_*·*n*H_2_O (**5**; 2D MOF) was synthesized using a typical hydrothermal approach.

## 2. Results and Discussion

### 2.1. Synthesis

All compounds were directly isolated from the solutions or contents of autoclaves mostly as good quality single crystals. The influence of several parameters, such as the type of metal centers, anions, inorganic/organic bases, ligand:metal ratio, volume of water, temperature and stirring in the formation and crystallization of the compounds was investigated. A number of drawbacks in the crystallization process were found:

(i) the use NaOH or triethylamine (Et_3_N) to deprotonate H_2_atrc in reactions with transition metal salts (such as Mn^2+^, Fe^2+^, Co^2+^, Ni^2+^, Cu^2+^, Zn^2+^ and Cd^2+^) led to poor crystalline powders;
(ii) the use of temperatures above 60 °C in the reactions with the aforementioned metals also led to the formation of microcrystalline powders;
(iii) with the use of less than *ca*. 6 mL of water in the systems mixed crystals of the new materials and the parent H_2_atrc ligand were easily produced;
(iv) with 30 min of stirring, the clear mixed solutions become cloudy and no pure single crystals were produced.

As a result of this study and optimization, the most adequate reaction conditions for each compound were ultimately optimized for a mixture of an aqueous solution of H_2_atrc and the metal, without stirring and standing at ambient temperature. The optimized conditions (see Experimental Section for details) were used in the reactions of H_2_atrc with several metal salts. The use of distinct Zn^2+^ salts [ZnCl_2_, Zn(NO_3_)_2_·6H_2_O, ZnSO_4_·7H_2_O and Zn(ClO_4_)·6H_2_O] with H_2_atrc at a ratio of 0.1:0.1–0.6 mmol (with a step of 0.1 mmol) resulted always in the isolation of the same complex **1**, indicating that this is a governing product under the fixed conditions. A similar phenomena was observed for the Mn^2+^ complexes, but the optimized reaction conditions are not suitable for the Cu^2+^, Ni^2+^ and Co^2+^, whose products appeared as powders [Cu^2+^ and Ni^2+^ series] or solutions [Co^2+^ series; slow evaporation of the solution did not result in suitable crystals], ultimately indicating that the metal centers play a decisive role in the formation of the final compounds.

While the slow evaporation process allowed the preparation of mononuclear ([Zn(Hatrc)_2_(H_2_O)] (**1**) and [Mn(Hatrc)_2_(H_2_O)_2_]·2H_2_O (**2**)) and dinuclear ([Fe_2_(Hatrc)_4_(OH)_2_]·6H_2_O (**3**)) complexes, the diffusion process and hydrothermal reaction conducted to the formation of 1D ([Cd(Hatrc)_2_(H_2_O)]*_n_* (**4**)) and 2D ([Mn(atrc)(H_2_O)]*_n_*·*n*H_2_O (**5**)) coordination networks.

### 2.2. Mononuclear Complexes

**Scheme 1:**
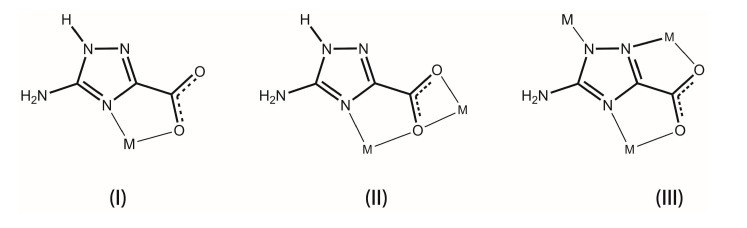
Coordination modes of Hatrc^−^ (type **I** and **II**) and atrc^2−^ (type **III**) anionic residues found in compounds **1**–**5**.

**Figure 1:**
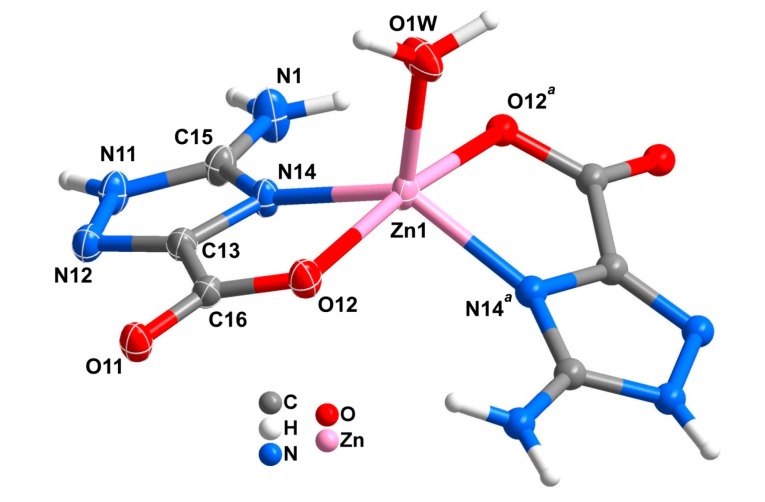
Schematic representation of the mononuclear complex [Zn(Hatrc)_2_(H_2_O)] present in compound **1**, showing the labelling scheme for all non-H atoms composing the asu and those forming the metal coordination sphere. Non-H atoms of the asu are drawn as thermal ellipsoids at the 30% probability level and the remaining atoms as spheres with arbitrary radius. For selected bond lengths and angles see Table 1. Symmetry transformation: *a* = *1*−*x*, *y*, *1.5*−*z*.

Complex **1**, [Zn(Hatrc)_2_(H_2_O)], crystallizes in the orthorhombic space group *Pbcn* with a crystallographic *C*_2_ rotation axis located along the Zn1–O1W bond. The metal center coordinates to one water molecule to two symmetry related Hatrc^−^ moieties, having a distorted trigonal bipyramidal coordination geometry whose triangular equatorial plane is formed by O1W, N14 and N14*^a^* (symmetry operation: *a* = *1*−*x*, *y*, *1.5*−*z*), while the axial positions are occupied by O12 and O12*^a^* atoms (Figure 1). The metal center is ideally located in the triangular plane with no deviation from the best least-squares plane fitted by the three coordinated atoms. The {ZnN_2_O_3_} trigonal bipyramid displays τ = (β − α)/60 = 0.6255, where α and β are the two largest angles found in the metal coordination center (Please note: τ = 0 for an ideal square pyramid, and τ = 1 for an ideal trigonal bipyramid) [48]. The Hatrc^−^ residue adopts a typical *N*,*O*-bidentate chelating mode (Scheme 1, type I) with the Zn–O and Zn–N found within the expected ranges (Table 1). Considering compounds **1**, **2** and the isotypical [Cd(Hatrc)_2_(H_2_O)_2_]·2H_2_O [34], the distances between the metal centers and the atoms in the first coordination sphere follow the tendency of the ionic radius Zn^2+^ < Mn^2+^ < Cd^2+^. Conversely, the O–M–N bite angles subtended by the Hatrc^−^ residue follow, as expected, the opposite tendency (Table 1).

**Figure 2:**
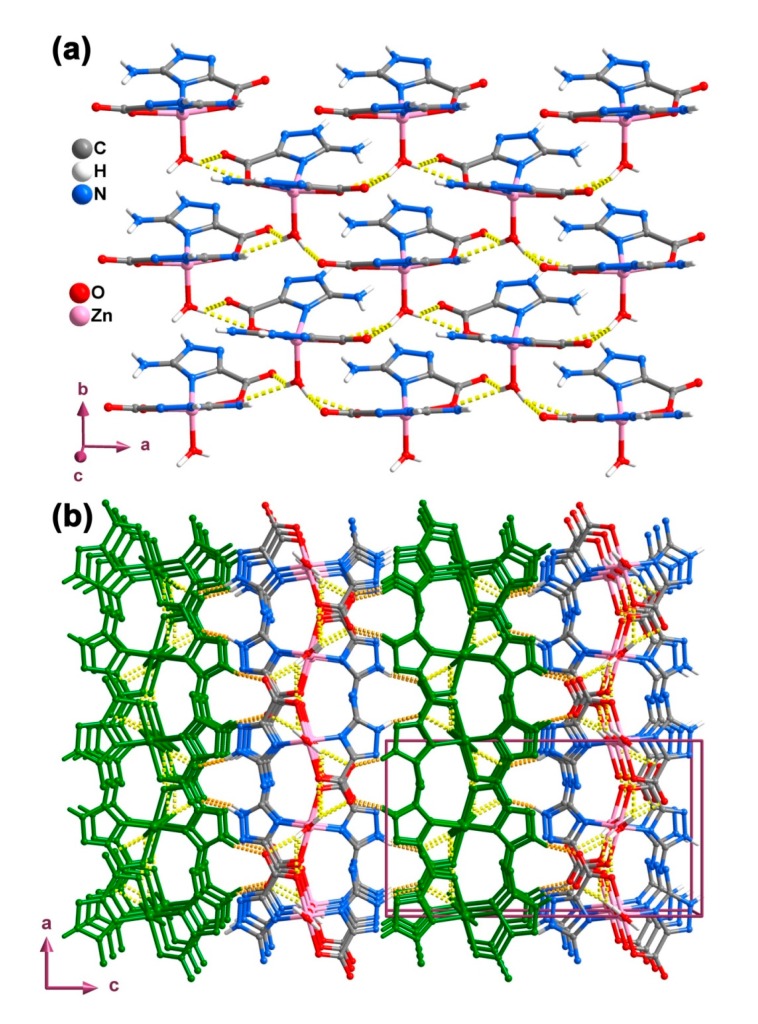
Ball-and-stick representation of (**a**) the 2D hydrogen-bonded network and (**b**) 3D hydrogen-bonded supramolecular network (layers with alternating colors) in the crystal structure of compound **1**. Hydrogen bonds are represented as yellow (those forming the 2D network) and orange (inter-layer connections) dashed lines. Geometric details on the represented interactions are listed in Table 2.

Adjacent [Zn(Hatrc)_2_(H_2_O)] complexes interact via strong hydrogen bonds leading to the formation of a 2D supramolecular structure (layers) extended in the *ab* plane of the unit cell (Figure 2a). The intermolecular hydrogen bonds involved in the formation of these layers are of the type O1W–H1WA···O11 and O1W–H1WA···O12 (yellow dashed lines in Figure 2a; Table 2). Neighboring layers pack close along the [001] direction and are interconnected into a 3D supramolecular architecture network via the N11–H11A···O11 interaction (orange dashed lines in Figure 2b; Table 2). Furthermore, the distance between the centroid of triazole ring and N11 is 3.510(3) Ǻ, pointing to the existence of a weak N11–H11A···π stacking interaction in the crystal structure (not shown), which also contributes for the stabilization of the 3D supramolecular architecture.

**Table 1:** Selected bond lengths (Å) and angles (°) for the metal coordination centers of mononuclear complexes [Zn(Hatrc)_2_(H_2_O)] (**1**), [Mn(Hatrc)_2_(H_2_O)_2_]·2H_2_O (**2**) and, for comparison, [Cd(Hatrc)_2_(H_2_O)_2_]·2H_2_O [34].

1		2		*Ref* [34]	
Zn1–O1W	1.975(5)	Mn1–O1W	2.206(2)	Cd1–O3	2.384(2)
Zn1–N14	1.981(3)	Mn1–N14	2.239(3)	Cd1–N1	2.255(3)
Zn1–O12	2.171(3)	Mn1–O1	2.186(2)	Cd1–O1	2.345(2)
N14–Zn1–O12	79.57(12)	O1–Mn1–N14	76.23(8)	O1–Cd1–N1	73.14(5)
O12–Zn1–O12 *^a^*	168.94(17)	O1–Mn1–O1 *^b^*	180.0		
O1W–Zn1–O12	95.53(9)	O1–Mn1–O1W	87.38(8)		
N14–Zn1–N14 *^a^*	131.5(2)	N14–Mn1–N14 *^b^*	180.0		
O1W–Zn1–N14	114.25(11)	O1W–Mn1–N14	90.44(8)		
		O1–Mn1–N14 *^b^*	103.77(8)		
		O1–Mn1–O1W *^b^*	92.62(8)		
		*O1W–Mn1–N14 ^b^*	*89.56(9)*		
		O1W–Mn1–O1W *^b^*	180.0		

Symmetry transformations used to generate equivalent atoms: (*a*) *1−x*, *y*, *1.5−z*; (*b*) *1−x*, *1−y*, *1−z*.

**Table 2:** Hydrogen-bonding geometrical details for the interactions present in the crystal structures of [Zn(Hatrc)_2_(H_2_O)] (**1**) and [Mn(Hatrc)_2_(H_2_O)_2_]·2H_2_O (**2**).

	D–H···A	D···A/Å	<DHA/°
**1**	N11–H11A···O11 *^c^*	2.749(4)	171
	N1–H1A···O12 *^a^*	3.191(5)	141
	N1–H1A···O1W *^d^*	3.322(4)	144
	N1–H1A···N12 *^c^*	2.964(5)	167
	O1W–H1WA···O11 *^e^*	2.781(3)	158
	O1W–H1WA···O12 *^e^*	3.031(3)	131
**2**	N11–H11A···O2W *^f^*	2.820(3)	142
	N12–H12A···O2W *^g^*	2.957(5)	116
	N1–H1A···O1W *^h^*	2.957(4)	173
	N1–H1B···O2W *^f^*	3.115(4)	142
	N1–H1B···O2W *^h^*	3.141(4)	128
	O1W–H1WA···O1 *^i^*	2.689(3)	160
	O1W–H1WB···O2 *^j^*	2.639(3)	162
	O2W–H2WB···O2 *^j^*	2.757(3)	175
	O2W–H2WA···N12 *^k^*	2.959(3)	152

Symmetry transformations: (*a*) *1*−*x*, *y*, *1.5*−*z*; (*c*) −*0.5*+*x*, *0.5*−*y*, *2*−*z*; (*d*) −*0.5*+*x*, −*0.5*+*y*, *1.5*−*z*; (*e*) −*0.5*+*x*, *0.5*+*y*, *1.5*−*z*; (*f*) −*x*, *1*−*y*, −*z*; (*g*) *x*, −*1*+*y*, *z*; (*h*) −*1*+*x*, *y*, *z*; (*i*) *2*−*x*, *1*−*y*, *1*−*z*; (*j*) *x*, *1*+*y*, *z*; (*k*) *1*−*x*, *1*−*y*, −*z*.

**Figure 3:**
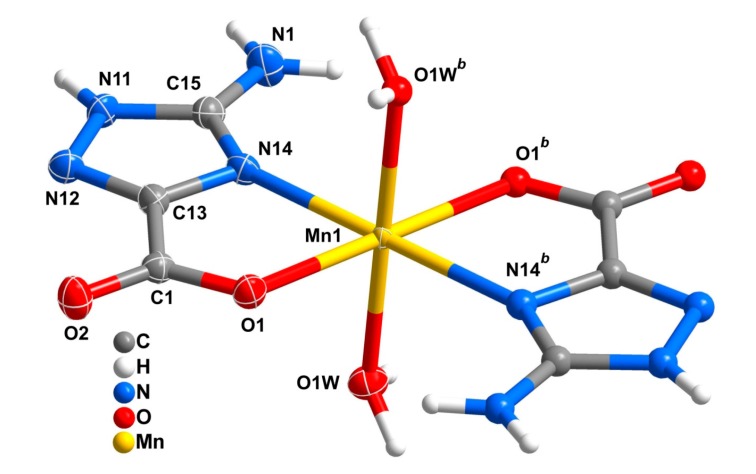
Mononuclear anionic complex [Mn(Hatrc)_2_(H_2_O)_2_] found in **2** showing the labelling scheme for all non-H atoms composing the asu and the metallic coordination environment. Crystallographically independent non-H atoms are represented as thermal ellipsoids drawn at the 50% probability level. Symmetry-related atoms are represented as spheres with arbitrary radius. For selected bond lengths and angles see Table 1. Symmetry operation: *b* = *1*−*x*, *1*−*y*, *1*−*z*.

Compound **2**, [Mn(Hatrc)_2_(H_2_O)_2_]·2H_2_O features a mononuclear complex (Figure 3) with the asu being composed of one metal center (Mn1), one Hatrc^−^ anionic ligand and one coordinated water molecule. An additional uncoordinated water molecule of crystallization is, however, present. The metal center has a distorted octahedron geometry, with the equatorial plane formed by O1, N14 and their symmetrical counterparts (O1*^b^* and N14*^b^*, symmetry transformation: *b*: *1*−*x*, *1*−*y*, *1*−*z*); O1W and O1W*^b^* occupy the axial positions (Figure 3 and Table 1). The coordination fashion of the ligand is of type I, as that observed in compound **1**. Bond-valence-sum (BVS) calculations gives a value of 2.02 for Mn1, indicating that Mn1 has the +2 oxidation state (command Calc Coord in PLATON [49]). This compound is isotypical to the already described compound [Cd(Hatrc)_2_(H_2_O)_2_]·2H_2_O [34].

**Figure 4:**
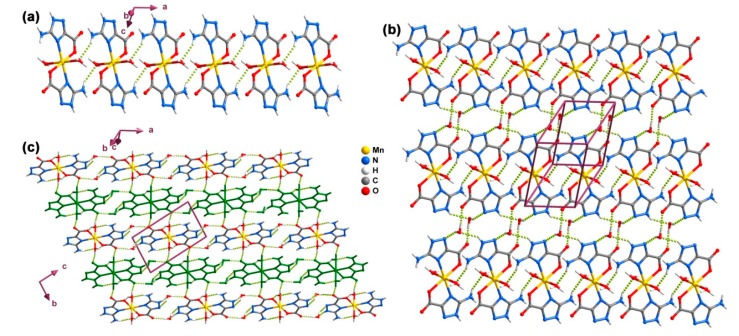
Ball-and-stick representation of (**a**) the 1D hydrogen-bonded chain; (**b**) the 2D layer and (**c**) the 3D supramolecular network (layers alternating between original and green color) present in compound **2**. Hydrogen bonds are represented by dashed light-green lines. For details on the represented interactions see Table 2.

Individual [Mn(Hatrc)_2_(H_2_O)_2_] complexes present in compound **2** are interconnected through strong hydrogen bonds of type N–H···O1W involving the coordinated water molecules and adjacent Hatrc^−^ residues into 1D chains running parallel to the *a*-direction of the unit cell (Figure 4a), forming a graph set motif of the type R_2_^2^(12) [50]. Chains further grow into supramolecular layers by way of the lattice water molecules O2W, which act as bridges between adjacent chains: O2W–H2WB···O2, O2W–H2WA···N12 and N11–H11A···O2W (Figure 4b and Table 3). Layers pack along the *b*-axis of the unit cell mediated by hydrogen bonds, ultimately originating the extended 3D supramolecular structure in **2** (Figure 4c).

**Table 3:** Selected bond lengths (Å) and angles (°) of the binuclear complex [Fe_2_(Hatrc)_4_(OH)_2_] present in compound **3**, and of the coordination networks [Cd(Hatrc)_2_(H_2_O)]*_n_* (**4**) and [Mn(atrc)(H_2_O)]*_n_*·*n*H_2_O (**5**).

3		4		5	
Fe1–O5	1.949(2)	Cd1–N24	2.239(5)	Mn1–N12 *^n^*	2.192(2)
Fe1–O5 *^l^*	1.979(2)	Cd1–N14	2.299(5)	Mn1–O1W	2.194(2)
Fe1–O4	2.030(2)	Cd1–O3	2.344(5)	Mn1–O1	2.2179(19)
Fe1–O1	2.041(3)	Cd1–O4 *^m^*	2.397(4)	Mn1–O2 *^o^*	2.2531(19)
Fe1–N14	2.096(3)	Cd1–O1W	2.407(5)	Mn1–N11 *^o^*	2.255(2)
Fe1–N24	2.113(3)	Cd1–O2	2.434(4)	Mn1–N14	2.299(2)
		Cd1–O3 *^m^*	2.593(4)		
O5–Fe1–O5 *^l^*	76.37(10)	N24–Cd1–N14	125.39(19)	N12 *^n^*–Mn1–O1W	88.23(8)
O5–Fe1–O4	165.65(10)	N24–Cd1–O3	73.44(17)	N12 *^n^*–Mn1–O1	100.41(8)
O5–Fe1–O1	92.88(10)	N24–Cd1–O4 *^m^*	140.30(17)	N12 *^n^*–Mn1–O2 *^o^*	168.57(8)
O5–Fe1–N14	97.93(10)	N24–Cd1–O1W	88.15(19)	N12 *^n^*–Mn1–N11 *^o^*	97.87(8)
O5–Fe1–N24	97.79(10)	N24–Cd1–O2	87.70(17)	N12 *^n^*–Mn1–N14	94.18(8)
O5 *^l^*–Fe1–O4	90.56(10)	N24–Cd1–O3 *^m^*	87.73(17)	O1W–Mn1–O1	94.82(7)
O5 *^l^*–Fe1–O1	164.41(10)	N14–Cd1–O3	88.21(18)	O1W–Mn1–O2 *^o^*	83.68(7)
O5 *^l^*–Fe1–N14	92.25(11)	N14–Cd1–O4 *^m^*	85.21(17)	O1W–Mn1–N11 *^o^*	98.93(8)
O5 *^l^*–Fe1–N24	95.89(11)	N14–Cd1–O1W	137.85(19)	O1W–Mn1–N14	170.92(9)
O4–Fe1–O1	101.08(10)	N14–Cd1–O2	70.68(17)	O1–Mn1–O2 *^o^*	88.34(7)
O4–Fe1–N14	88.36(10)	N14–Cd1–O3 *^m^*	127.60(17)	O1–Mn1–N11 *^o^*	157.40(8)
O4–Fe1–N24	77.40(10)	O3–Cd1–O4 *^m^*	138.78(15)	O1–Mn1–N14	76.15(8)
O1–Fe1–N14	77.89(11)	O3–Cd1–O1W	77.05(17)	O2 *^o^*–Mn1–N11 *^o^*	75.53(8)
O1–Fe1–N24	96.75(11)	O3–Cd1–O2	135.96(16)	O2 *^o^*–Mn1–N14	95.06(8)
N14–Fe1–N24	163.63(11)	O3–Cd1–O3 *^m^*	143.57(3)	N11 *^o^*–Mn1–N14	89.42(8)
		O4 *^m^*–Cd1–O1W	80.97(16)		
		O4 *^m^*–Cd1–O2	79.08(15)		
		O4 *^m^*–Cd1–O3 *^m^*	52.60(14)		
		O1W–Cd1–O2	143.05(16)		
		O1W–Cd1–O3 *^m^*	71.32(16)		
		O2–Cd1–O3 *^m^*	71.83(15)		

Symmetry transformations used to generate equivalent atoms: (*l*) *x*, *0.5*−*y*, *0.5*−*z*; (*m*) *1.5*−*x*, *0.5+y*, *0.5*−*z*; (*n*) −*x*, *0.5+y*, *0.5*−*z*; (*o*) *x*, *0.5*−*y*, *z*−*0.5*.

### 2.3. Binuclear Complex

**Figure 5:**
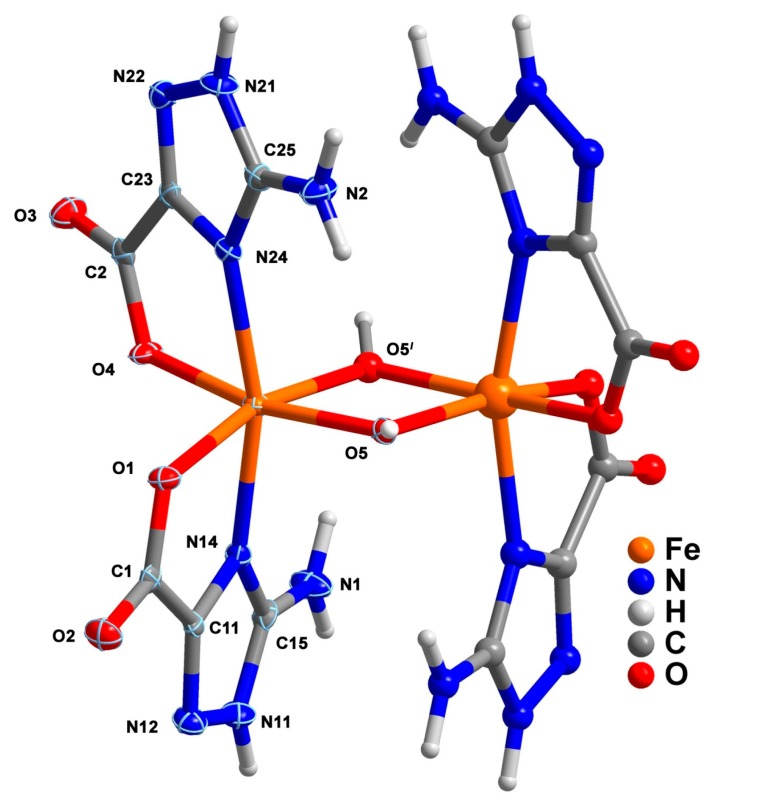
Representation of the binuclear complex [Fe_2_(Hatrc)_4_(OH)_2_] present in compound **3**, showing the labelling scheme for all non-H atoms composing the asu and the Fe1 coordination environment. Non-H atoms of the asu are represented as thermal ellipsoids (70% probability level) and the remaining atoms as spheres with arbitrary radii. For selected bond lengths and angles see Table 3. Symmetry operation: *l* = *x*, *0.5*−*y*, *0.5*−*z.*

Compound **3**, [Fe_2_(Hatrc)_4_(OH)_2_]·6H_2_O, is composed by a discrete neutral binuclear unit (Figure 5). The asu is composed of one independent Fe^3+^ center, two anionic Hatrc^−^ ligands, one μ_2_-bridging OH^−^ anion and three lattice water molecules. Besides the presence of the aforementioned anions, the charge of the metallic center is supported by Bond Valence Sum (BVS) calculations, which produces a value for the ferric atom of *ca.* 3.01, thereby indicating that iron center is indeed in the +3 oxidation state [49]. The six-coordinated Fe1 center displays a distorted octahedral coordination geometry with the apical positions being occupied by N14 and N24 from two coordinated triazole moieties. The carboxylic oxygen atoms O2 and O4 from two distinct carboxylate groups plus O5 and O5*^l^* from two crystallographic equivalent hydroxyl moieties shape the equatorial plane of the octahedron (symmetry transformation to generate equivalent atoms: *l = x*, 0.5−*y*, 0.5−*z*). The resulting binuclear [Fe_2_(Hatrc)_4_(OH)_2_] complex is thus formed due two μ_2_-OH^−^ bridging anions, with the Fe1–O5 distances being 1.954(2) and 1.985(3) Ǻ, well within the expected values for Fe–O distances in the Fe–OH–Fe moiety [1.936–2.085 Å, CSD version 5.36, updated in November 2014, 17 observations], and imposing an internuclear Fe1···Fe1*^l^* separation of 3.087(1) Ǻ (Figure 5 and Table 3 for geometric details on selected bond lengths and angles). It is noteworthy that the coordination mode of Hatrc^−^ found in **3** is identical to that described for compounds **1** and **2**.

**Figure 6:**
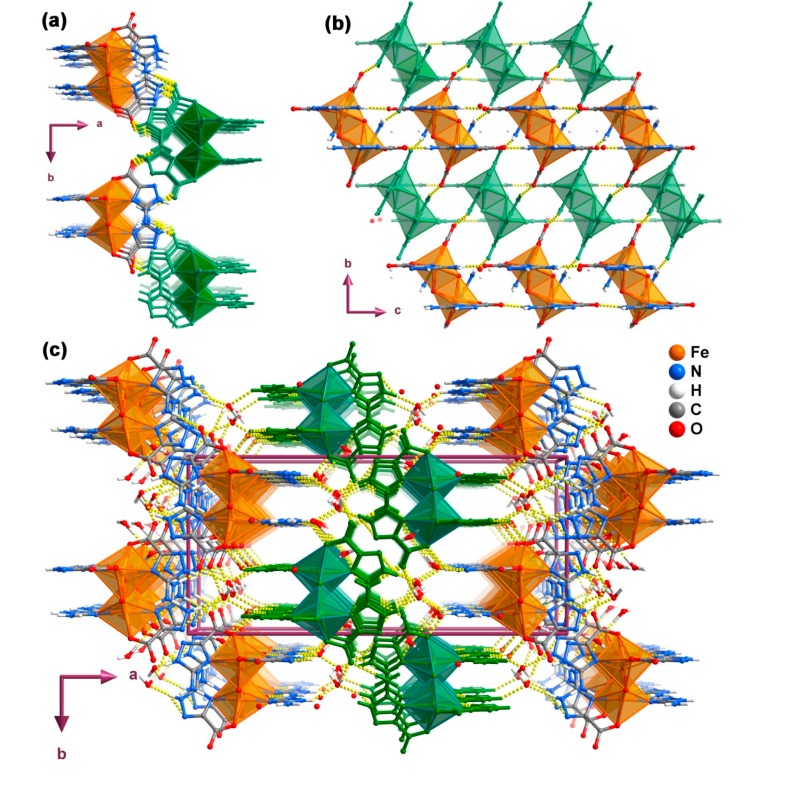
Ball-and-stick representation of the 2D hydrogen-bonded layer present in compound **3** viewed along the (**a**) [001] and (**b**) [100] directions of the unit cell (1D supramolecular chains represented in alternate colors); (**c**) Perspective view of the 3D supramolecular framework (layers alternate between colors) present in the crystal structure of **3**. Dashed yellow lines represent the hydrogen bonds. Geometric details on these supramolecular interactions are summarized in Table 4.

Supramolecular interactions are of two main types: those involving solely individual Fe^3+^ complexes and those that interconnect complexes with lattice water molecules. Neighboring [Fe_2_(Hatrc)_4_(OH)_2_] complexes are interconnected through strong hydrogen bonds of the type N–H···O into 1D supramolecular chains that run parallel to the *c*-direction of the unit cell. 2D supramolecular layers placed in the *bc* plane of the unit cell are formed by hydrogen bonds of type N1–H1B···O2 (Figure 6a,b; Table 4 for geometrical details on the hydrogen bonds). Adjacent layers are fused into a 3D supramolecular architecture by ways of several strong N–H···O, O–H···N and O–H···O interactions (Figure 6c and Table 4).

**Table 4:** Geometrical details on the hydrogen bonds present in compound [Fe_2_(Hatrc)_4_(OH)_2_]·6H_2_O (**3**).

D–H···A	D···A/Å	<DHA/°	D–H···A	D···A/Å	<DHA/°
N11–H11A···O2W	2.746(4)	171	O1W–H1WA···O3W^*u*^	2.847(4)	174
N1–H1A···O3W *^p^*	2.905(4)	151	O1W–H1WB···N22^*t*^	2.914(4)	179
N1–H1B···O2 *^q^*	3.023(4)	173	O2W–H2WA···O1W^*v*^	2.678(4)	154
N21–H21A···O2W *^r^*	2.903(4)	135	O2W–H2WB···O2 *^q^*	2.842(4)	145
N21–H21A···O1 *^s^*	2.866(4)	128	O3W–H3WA···O4	2.719(3)	166
N2–H2A···O3 *^t^*	2.914(4)	163	O3W–3WB···N12 *^k^*	2.830(4)	175
N2–H2B···O1W	2.872(4)	154	O5–H5A···O3 *^t^*	2.829(3)	159

Symmetry transformations used to generate equivalent atoms: (*k*) *1−x*, *1−y*, *−z*; (*p*) *x*, *0.5−y*, *−0.5−z*; (*q*) *1−x*, *y−0.5*, *z−0.5*; (*r*) *0.5+x y*, *−z*; (*s*) *1.5−x*, *1−y*, *z*; (*t*) *x*, *y*, *1+z*; (*u*) *1.5−x*, *1−y*, *1+z*; (*v*) *0.5−x*, *0.5−y*, *0.5−z*.

### 2.4. One-Dimensional Chain (1D)

**Figure 7:**
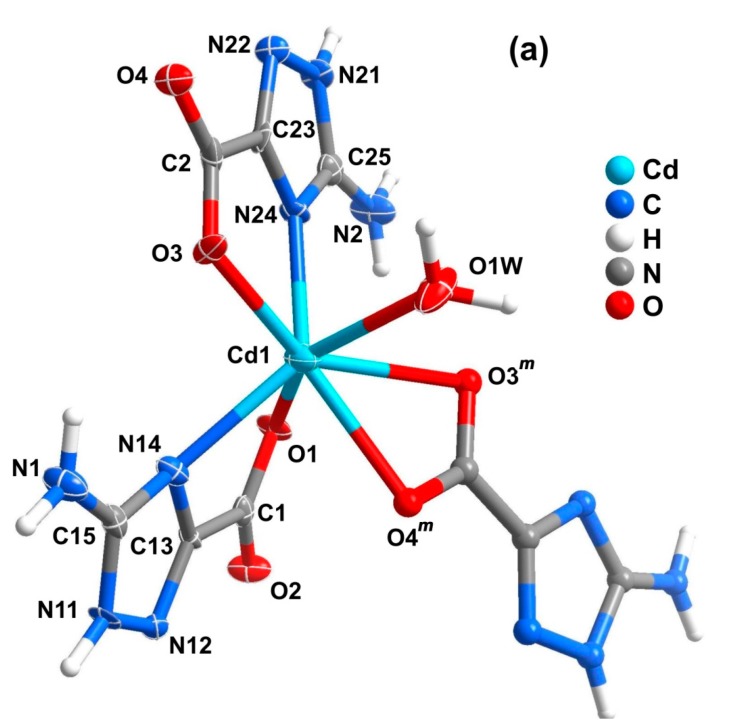

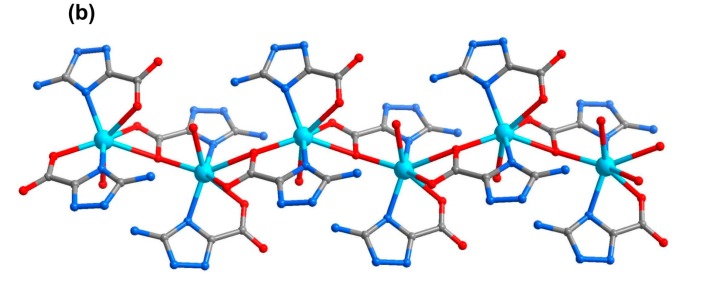
Schematic representation of the (**a**) Cd^2+^ coordination environment present in compound [Cd(Hatrc)_2_(H_2_O)]*_n_* (**4**) showing the labeling scheme for all non-hydrogen atoms composing the asu and the Cd1 coordination environment. Non-H atoms of the asu are represented as thermal ellipsoids drawn at the 70% probability level and the remaining atoms as spheres with arbitrary radii; (**b**) 1D coordination chain present in compound **4**. For selected bond lengths (in Å) and angles (in degrees) see Table 3. Symmetry operation: *m* = *1.5*−*x*, *y+0.5*, *0.5*−*z.*

Compound **4**, [Cd(Hatrc)_2_(H_2_O)]*_n_*, crystallizes in monoclinic space group *P*2_1_/*n* with one Cd^2+^ cation, two Hatrc^−^ ligands and one coordinated water composing the asu, which features the building unit of a 1D coordination chain (Figure 7). The Cd1 center coordinates to three Hatrc^−^ anionic ligands and one water molecule (Figure 7a), displaying a coordination geometry which resembles a relatively rare seven-coordinated capped trigonal prism: the atom groups N14, O2, O4 and N24, O3 and O1W build the two opposite triangular facets, and another symmetrical O3 is the capping atom located at the rectangular facet fitted by O2, O4, N24 and O1W (selected bond distances and angles are in given in Table 3). Although seven-coordinated Cd^2+^ centers are not so numerous as those of four or six-coordinated, a search in the CCDC [33] gives up to 700 related hits. Limiting the search to 1,2,4-triazole residues only, 33 examples of seven-coordinated cadmium(II) are known: three of them are single-capped octahedra [48,51,52,53] while the remaining ones are pentagonal bipyramids. The two crystallographically distinct Hatrc^−^ ligands in compound **4** adopt two different coordination fashions: one is a η^2^-type (type I in Scheme 1) and the other is η^4^-type (type II in Scheme 1). The first coordination mode is the same as those observed in compounds **1**–**3**, chelating to the metal center by one carboxylate O-atom and the 4-positioned N-atom of the triazole ring to form a five-membered ring. The η^4^-type Hatrc^−^ ligand adopts both the *N*,*O*-bidente chelating mode described previously, and *O*,*O*-bidente-chelating mode to bind two adjacent Cd^2+^ centers: two O-atoms of the carboxylate group chelate to one metal center to form a four-membered ring. As a consequence of the aforementioned linkages of the η^4^-Hatrc^−^ ligand, the neighboring Cd1 centers are interconnected into wave-like 1D [Cd(Hatrc)]*_n_* chains running parallel to the *b*-direction of the unit cell and comprising two kinds of five and four-membered rings. The η^2^-Hatrc^−^ ligands connect Cd1 centers on both sides of the chain. This, alongside with the coordinated water molecules, ultimately form the 1D coordination polymer (chain) [Cd(Hatrc)_2_(H_2_O)]*_n_* (Figure 7b).

**Figure 8:**
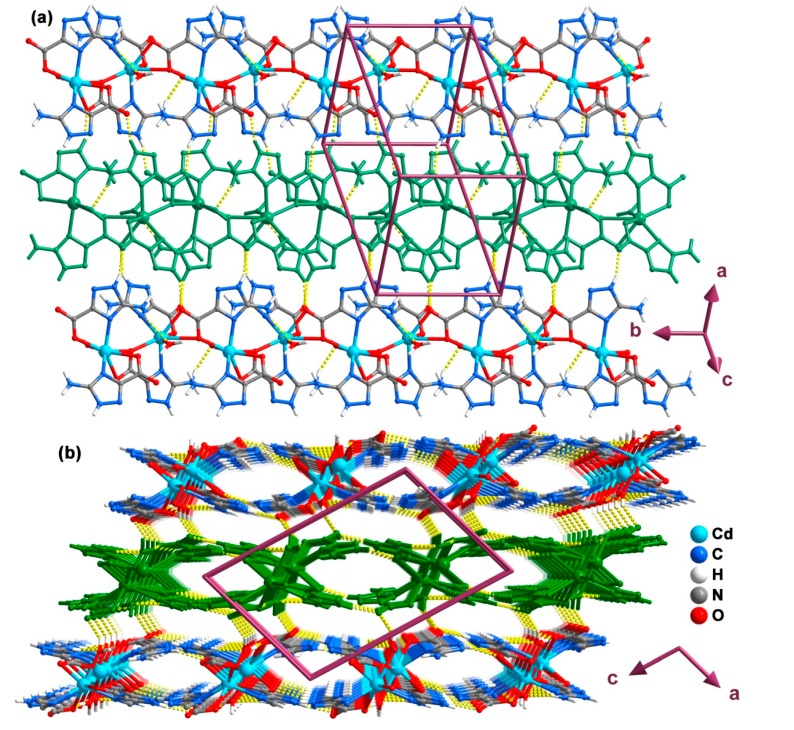
Ball-and-stick representation of the (**a**) 2D hydrogen-bonded network formed between adjacent 1D coordination chains (represented with alternating colors) and the (**b**) 3D hydrogen-bonded supramolecular network viewed in perspective along the [010] direction of the unit cell (layers represented with alternating colors) present in compound **4**. Hydrogen bonds are represented as dashed yellow lines. Geometric details on these interactions are summarized in Table 5.

Neighboring chains interact trough cooperative N–H···O hydrogen bonds, in particular interactions of the type N11–H11A···O4, N2–H2A···O1W and N21–H21A···O1, leading to the formation of 2D supramolecular layers as depicted in Figure 8a (see Table 5 for geometrical details on the existent hydrogen bonds). Additionally, these supramolecular layers are interconnected to other adjacent ones via strong N–H···O and O–H···O interactions forming a 3D supramolecular architecture (Figure 8b and Table 5).

**Table 5:** Geometrical details on the hydrogen bonds present in the supramolecular networks [Cd(Hatrc)_2_(H_2_O)]*_n_* (**4**) and [Mn(atrc)(H_2_O)]*_n_*·*n*H_2_O (**5**).

	D–H···A	D···A/Å	<DHA/°		D–H···A	D···A/Å	<DHA/°
**4**	N11–H11A···O4 *^w^*	2.769(7)	169	**5**	N1–H1A···O *^A^*	2.966(3)	156
	N1–H1A···O1W *^x^*	3.200(8)	162		N1–H1B···O1W *^f^*	3.224(3)	151
	N1–H1B···N22 *^w^*	3.211(8)	153		O1W–H1WA···O2 *^b^*	2.748(3)	161
	N21–H21A···O1 *^y^*	2.684(6)	164		O1W–H1WB···O2W *^B^*	2.714(3)	152
	N2–H2A···O1W *^m^*	3.064(8)	155		O2W–H2WB···N1	2.872(3)	160
	N2–H2B···N12 *^y^*	3.037(8)	158		O2W–H2WA···N14 *^C^*	3.186(3)	138
	O1W–H1WB···O1 *^z^*	2.747(7)	165		O2W–H2WA···N12 *^D^*	3.183(3)	129
	O1W–H1WA···O2 *^x^*	2.698(7)	158		O2W–H2WA···N11 *^D^*	3.159 (3)	121

Symmetry transformations used to generate equivalent atoms: (*b*) *1−x*, *1−y*, *1−z*; (*f*) *−x*, *1−y*, *−z*; (*m*) *1.5−x*, *0.5+y*, *0.5−z*; (*w*) *0.5+x*, *−0.5−y*, *z−0.5*; (*x*) *1.5−x*, *−0.5+y*, *0.5−z*; (*y*) *0.5+x*, *0.5−y*, *0.5+z*; (*z*) *0.5+x*, *0.5−y*, *0.5+z*; (*A*) *−1+x*, *0.5−y*, *−0.5+z*; (*B*) *1+x*, *0.5−y*, *0.5−z*; (*C*) *x*, *0.5−y*, *z−0.5*; (*D*) *−x*, *−y*, *−z*.

### 2.5. Coordination Layer (2D)

**Figure 9:**
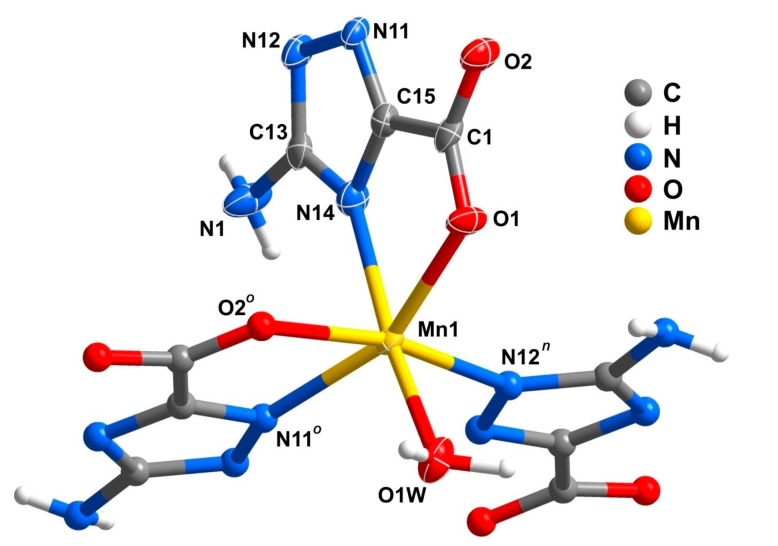
Mixed ellipsoid and ball-and-stick representation of the Mn^2+^ coordination environment present in compound [Mn(atrc)(H_2_O)]·H_2_O (**5**), showing the labeling scheme for all non-H atoms composing the asu and the first metal coordination sphere. Non-H atoms of the asu are represented as thermal ellipsoids drawn at the 90% probability level and the remaining atoms as spheres with arbitrary radius. For selected bond lengths and angles see Table 3. Symmetry operations: *n* = −*x*, *0.5+y*, *0.5−z*; *o* = *x*, *0.5−y*, −*0.5+z*.

The structure of compound **5**, as unveiled from single-crystal XRD analysis, was formulated as [Mn(atrc)(H_2_O)]·H_2_O, featuring a 2D coordination layered structure. The asu comprises one Mn^2+^ center, one fully deprotonated atrc^2−^ anionic ligand, one coordinated and one lattice water molecule. The Mn1 metal center is coordinated by three crystallographic equivalent atrc^2−^ and one water molecule, leading to a six-coordinated coordination environment with a geometry that resembles a distorted octahedron: N14, N12*^n^*, O2*^o^* and O1W atoms form the equatorial plane, and O1 and N11*^o^* occupy the two axial positions (Figure 9; for details concerning bond lengths and angles see Table 3; symmetry operations: *n* = −*x*, *0.5+y*, *0.5*−*z* and *o* = *x*, *0.5*−*y*, −*0.5+z*). BVS calculations gave a charge of +2.02 for Mn center suggesting that the oxidation state of Mn1 should be +2, in good agreement with the charge assignment from the crystal structure determination.

**Figure 10:**
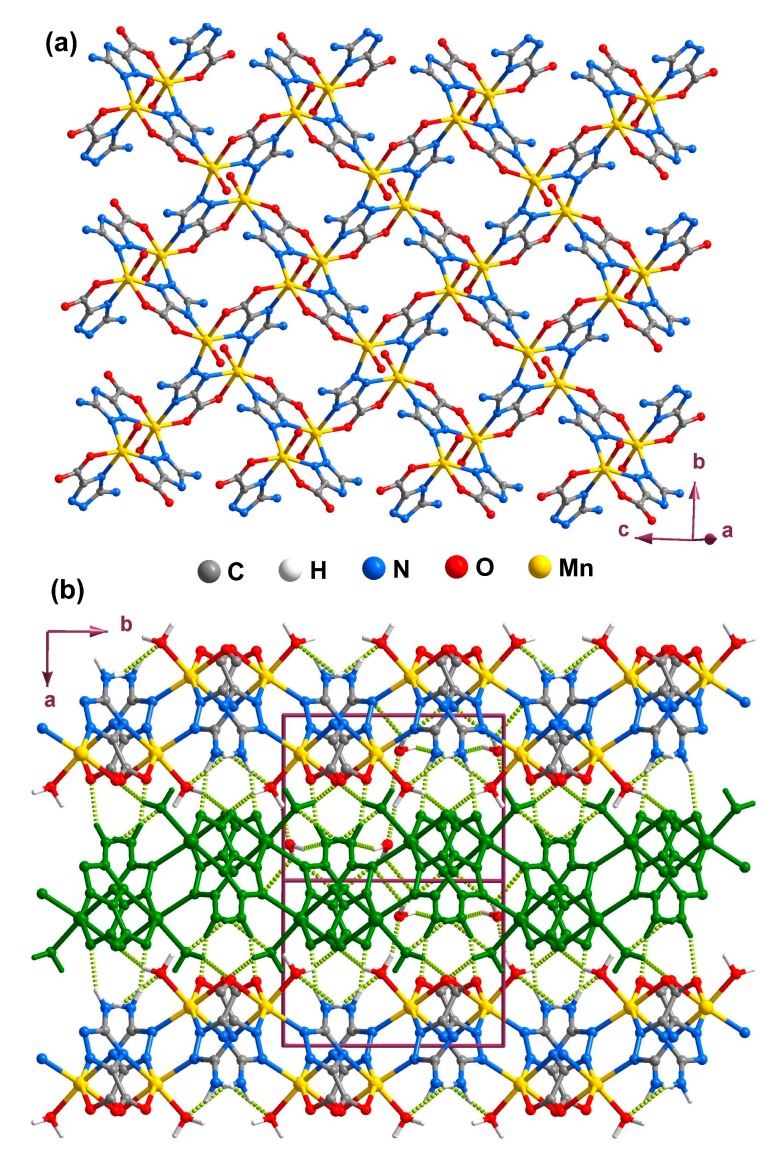
Ball-and-stick representation of (**a**) the 2D coordination layer and (**b**) 3D hydrogen-bonded network viewed along the [001] direction of the unit cell of compound **5** (layers represented with alternating colors). Hydrogen bonds are represented as dashed light-green lines. Geometric details on the represented supramolecular interactions are listed in Table 5.

Each atrc^2−^ residue in **5** connects to three Mn^2+^ metal centers through two *N*,*O*-bidentate interactions (chelating two metal cations through N11/O2 and N14/O1) and one monodentate-*N* mode to bind the fourth Mn^2+^ by N12 (Scheme 1, type III). As a consequence of these coordination modes, a 2D coordination framework (layer) based on the interconnection of [Mn_8_(atrc)_8_] macro rings is ultimately (Figure 10a). The 2D layers pack along the [100] direction of the unit cell being mediated by numerous strong O–H···O, O–H···N and N–H···O hydrogen-bonding interaction leading to the formation of a 3D supramolecular architecture (Figure 10b).

The coordination fashions of the organic ligand (Scheme 1) can be related with the structural features and dimensionality of the reported complexes. In mild conditions, H_2_atrc tends to firstly deprotonate the hydrogen atom of the carboxylic acid group. The resulting anionic Hatrc^−^ ligand chelates one metal center using the 4-positioned N-atom and one O-atom of the carboxylate group to form a rather stable five-membered chelate ring (type I in Scheme 1), as observed in compounds **1**–**3**. The coordination fashion of Hatrc^−^ in compound **4** (type II in Scheme 1) has one more chelating carboxylate group than type I. Usually, a more complex coordination fashion tends to promote more complicated structures. With the use of a higher synthetic temperature and the presence of a strong base (NaN_3_ or NaOH), the hydrogen atom on the 1-positioned N is liberated, thereby giving rise to a bidentate-chelating-*N*,*O* and one monodentate-*N* coordination donor (type III) contributing to the construction of a higher dimensional network.

## 3. Experimental Section

### 3.1. Materials and Methods

Most of the reagents and solvents were used directly as supplied commercially without further purification: 3-amino-1*H*-1,2,4-triazole-5-carboxylic acid (98%, Sigma-Aldrich, St-Louis, MO, USA, 4,4′-bipyridine hydrate (Sigma-Aldrich, , 98%), ZnSO_4_·6H_2_O (99.5%+, Merck, Kenilworth, NJ, USA), MnCl_2_·4H_2_O (Sigma-Aldrich, 99.0%+), Mn(OAc)_2_·4H_2_O (Sigma-Aldrich, 99.0%+), CdBr_2_·4H_2_O (98.0%, Sigma-Aldrich), Fe(NO_3_)_3_·9H_2_O (99.0%+, Merck), NaN_3_ (99.0%+, Sigma-Aldrich). Microcrystalline materials of Mn(ClO_4_)_2_·6H_2_O and Cd(ClO_4_)_2_·6H_2_O were isolated by freezing a reaction solution of HClO_4_ with excess of MnCO_3_ or CdCO_3_, respectively. (**Caution**: handling of perchlorate in solution or in the dry state should be performed in very small amounts and very carefully because these complexes are explosive in contact with organic or other readily oxidizable substances).

### 3.2. Synthesis

*[Zn(Hatrc)_2_(H_2_O)]* (**1**). H_2_atrc (4.992 mmol, 0.6394 g) was dissolved in 500 mL of hot water, cooled to ambient temperature and then mixed with a 5 mL ZnSO_4_·7H_2_O (5.019 mmol, 1.4404 g) aqueous solution without stirring. Single crystals suitable for X-ray diffraction were obtained from the standing solution over a period of two weeks. FT-IR (KBr): 3435 (νOH), 3327 (νN–H(···O)), 3220 (νN–H(···O), νNH_2_), 3121 (νNH_2_), 2940 (νNH, νCH), 2792 1656 (νO–H(···N)), 1599 (νC=O), 1535 (νC=N), 1477 1306 (νC–O), 1120 (δCH, νrg), 1063 820 (γO–H(···N)), 763 727 (γO–H, ρCH), 642 (γrg, νCN), 491 (ωNH_2_), 399 (δC–O) cm^−1^. FT-Raman: 3435 (νN–H(···O)), 3285 (νN–H(···O), νNH_2_), 3220 (νNH_2_), 3142, 1656 (νC=O), 1535, 1499, 1456, 1434 (δNH, νrg), 1378 (νC–O), 1299 (δCH, νrg), 1106 (γCNH, γrg, νCN (ring)), 1070 (νC–O, νC–C), 1042 (γO–H(···N)), 813 (γO–H(···N), γCNH, γNH_2_, ϕrg, ωCN), 756 (γO–H, ρCH), 491 (δC–O), 392 (τNH_2_), 199 (νM–N, ϕ(out of plane)NH_2_, γNCN), 149, 120 cm^−1^.

*[Mn(Hatrc)_2_(H_2_O)_2_]·2H_2_O* (**2**). A similar procedure to that previously described for **1** was employed: 5 mL of a MnCl_2_·4H_2_O (5.005 mmol, 0.9904 g) aqueous solution and 500 mL of a H_2_atrc (5.003 mmol, 0.6408 g) aqueous solution promoted the formation of colorless block single crystals. FT-IR (KBr): 3306 (νN–H(···O)), 3170 (νNH2), 3020 (νNH, νCH), 2784 1685 (νO–H(···N)), 1628 (νC=O), 1556 (νC=N), 1499 1363 (δNH, νrg), 1249 (νC–O), 1113 (δCH, νrg), 1049 (νC–O, νC–C), 820 (γO–H(···N)), 777 699 (γrg, νCN), 627 (ρCOO), 463 (ωNH_2_), 399 (δC–O) cm^−1^. FT-Raman: 3177, 3020 (νNH, νCH), 1685 (νC=O), 1553, 1506 (νrg, δCH, δNH), 1420, 1363 (νC–O), 1242 (δCH, νrg), 1113 (γCNH, γrg, νCN (ring)), 1049 (γO–H(···N)), 799 (γO–H(···N), γCNH, γNH_2_, ϕrg, ωCN), 727 (γrg, νCN), 570 (ωNH_2_), 463 (δC–O), 399 (τNH_2_), 227 (νM–N), 192 (νM–N, ϕ(out of plane)NH_2_, γNCN), 127, 99 cm^−1^.

*[Fe(Hatrc)_2_(OH)]_2_·6H_2_O* (**3**). An identical experimental procedure as that described for **1** was employed: 4.9985 mmol, (2.0194) g of Fe(NO_3_)_3_·9H_2_O (5 mL) with 5.004 mmol, 0.6410 g of H_2_atrc (500 mL) produced orange block single crystals. FT-IR (KBr): 3392 (νN–H(···O)), 3258 (νN–H(···O), νNH2), 3195 (νNH_2_), 2941 (νNH, νCH), 2772 1650 (νO–H(···N)), 1551 (νC=N), 1459 1354 (δNH, νrg), 1269 (νC–O), 1013 (δCH, νrg), 909 (νC–O, νC–C), 839 (γO–H(···N)), 754 726 (γO–H, ρCH), 655 (γrg, νCN), 592 (ρCOO), 458 (ωNH2), 402 (δC–O) cm^−1^. FT-Raman: 3392 (νN–H(···O), νNH_2_), 3329 (νNH_2_), 3181, 1643 (νC=O), 1544, 1466, 1410 (δNH, νrg), 1376 (νC–O), 1114 (γCNH, γrg, νCN (ring)), 754 (γO–H, ρCH), 564 (ωNH_2_), 500 (δC–O), 402 (τNH2), 303 (νM–N), 162, 112 cm^−1^.

*[Cd(Hatrc)_2_(H_2_O)]_n_* (**4**). 5 mL of a mixture of MeOH and H_2_O (v:v = 1:1) was gently added dropwise onto 9 mL of an aqueous solution containing H_2_atrc (0.100 mmol, 0.0128 g) and CdBr_2_·4H_2_O (0.0999 mmol, 0.0344 g). 8 mL of a MeOH solution of 4,4′-bipyridine (0.0948 mmol, 0.0148 g) was added carefully as a third layer. Colorless block single crystals were obtained on the inner wall of the glass tube after a one week diffusion process. FT-IR (KBr): 3363 (νN–H(···O)), 3235 (νN–H(···O), νNH2), 3142 (νNH_2_), 2970 (νNH, νCH), 2799 1670 (νO–H(···N)), 1620 (νC=O), 1528 (νC=N), 1477 1370 (δNH, νrg), 1292 (νC–O), 1128 (δCH, νrg), 1070 806 (γO–H(···N)), 756 713 (γO–H, ρCH), 663 (γrg, νCN), 620 (ρCOO), 477 (ωNH_2_), 413 (δC–O) cm^−1^. FT-Raman: 3370 (νN–H(···O), νNH_2_), 3135, 3070 (νNH, νCH), 1606 (νC=N), 1528, 1485, 1434 (δNH, νrg), 1385 (νC–O), 1292 (δCH, νrg), 1235, 1106 (γCNH, γrg, νCN (ring)), 1070 (νC–O, νC–C), 1013 (γO–H(···N)), 856 (γO–H(···N), γCNH, γNH_2_, ϕrg, ωCN), 763 (γO–H, ρCH), 656 (ρCOO, ), 563 (ωNH_2_), 484 (δC–O), 406 (τNH2), 270, 220 (νM–N), 199 (νM–N, ϕ(out of plane)NH_2_, γNCN), 127 cm^−1^.

*[Mn(atrc)(H_2_O)]_n_·nH_2_O* (**5**). *Method 1:* H_2_atrc (0.602 mmol, 0.0771 g) was treated with NaOH (0.6 mmol, 0.0240 g) in 15 mL of water with stirring at ambient temperature for 30 min to get a clear colorless solution. This solution was mixed with Mn(ClO_4_)_2_·6H_2_O (0.199 mmol, 0.0722 g) in a 25 mL Teflon-lined stainless-steel reaction vessel. The mixture was heated at 100 °C for 5 days in a furnace and then cooled to obtain colorless crystals of **5**. The same product can be obtained from the similar procedure using MnCl_2_·4H_2_O or Mn(OAc)_2_·4H_2_O instead of Mn(ClO_4_)·6H_2_O.

*Method 2:* H_2_atrc (0.400 mmol, 0.0512 g) mixed with NaN_3_ (0.400 mmol, 0.0260 g) in 18 mL of water was stirred at ambient temperature for 30 min to get a clear colorless solution, which was mixed with MnCl_2_·4H_2_O (0.205 mmol, 0.0406 g) in a 25 mL Teflon-lined stainless steel reaction vessel. The mixture was heated at 100 °C for 5 days in a furnace and then cooled slowly to ambient temperature to isolate a colorless crystalline material of compound **5**. FT-IR (KBr): 3375 (νN–H(···O)), 3240 (νN–H(···O), νNH_2_), 1620 (νC=O), 1505 (νC=N), 1318 (νC–O), 1106 (δCH, νrg), 849 (γO–H(···N)), 746 656 (γrg, νCN), 489 (ωNH2), 425 (δC–O) cm^−1^.

### 3.3. Single Crystal X-ray Diffraction

Single crystals of compounds **1**–**5** were manually harvested from the crystallization vials and mounted on Hampton Research CryoLoops using FOMBLIN Y perfluoropolyether vacuum oil (LVAC 25/6, purchased from Aldrich) [54] with the help of a Stemi 2000 stereomicroscope equipped with Carl Zeiss lenses. Data were collected with a Bruker X8 Kappa APEX II charge-coupled device (CCD) area-detector diffractometer (Mo-Kα graphite-monochromated radiation, λ = 0.71073 Å) controlled by the APEX2 software package [55], and equipped with an Oxford Cryosystems Series 700 cryostream monitored remotely using Cryopad [56]. Images were processed using SAINT+ [57], and data were corrected for absorption by the multi-scan semi-empirical method implemented in SADABS [58]. The structures of compounds **1**–**5** were solved by direct methods using SHELXS [59] and refined using SHELXL [60] by full-matrix least-squares technique on *F*^2^. All non-hydrogen atoms were refined anisotropically. Hydrogen atoms attached to C atoms were placed at geometrically calculated positions to their carrier atoms and refined with isotropic thermal parameters included in the final stage of the refinement, with *U*_iso_ = 1.2 × *U*_eq_ of the atoms which they are attached. Hydrogen atoms associated with the water molecules and nitrogen atoms were located in difference Fourier maps. The N–H, O–H and H···H distances were fixed using with *U*_iso_ = 1.5 × *U*_eq_ of the atoms to which they are attached, plus appropriated DFIX distances (N–H 0.88 Å, O–H 0.95 Å and H···H 1.54 Å). A summary of the structural determination and refinement details for compounds **1**–**5** is listed in Table 6. The highest peaks and deepest holes found in the structures refinement is listed in Table 7. CCDC 1403898-1403902 contain the supplementary crystallographic data for this paper. These data can be obtained free of charge from The Cambridge Crystallographic Data Centre via www.ccdc.cam.ac.uk/data_request/cif.

**Table 6:** Crystal and structure refinement data for compounds **1**–**5**.

	1	2	3	4	5
Empirical formula	C_6_H_8_N_8_O_5_Zn	C_6_H_14_MnN_8_O_8_	C_12_H_26_Fe_2_N_16_O_16_	C_6_H_8_CdN_8_O_5_	C_3_H_6_MnN_4_O_4_
Color and Habit	Colorless Plate	Colorless block	Orange block	Colorless block	Colorless block
Crystal Size (mm^3^)	0.42 × 0.21 × 0.07	0.08 × 0.04 × 0.03	0.07 × 0.02 × 0.02	0.07 × 0.03 × 0.04	0.12 × 0.11 × 0.05
Crystal system	Orthorhombic	Triclinic	Orthorhombic	Monoclinic	Monoclinic
Space group	*Pbcn*	*P*ī	*Pnna*	*P*2_1_/*n*	*P*2_1_/*c*
*a* (Ǻ)	9.537(3)	5.2482(9)	27.111(6)	8.9893(13)	8.1472(6)
*b* Ǻ	6.865(2)	6.5330(11)	12.697(3)	8.9785(13)	9.9846(7)
*c* (Ǻ)	17.120(5)	10.6128(18)	7.7352(17)	13.9642(16)	9.3679(6)
α (°)	90	90.532(10)	90	90	90
β (°)	90	102.774(11)	90	108.117(5)	113.994(4)
γ (°)	90	109.139(10)	90	90	90
*V* (Ǻ^3^)	1120.8(6)	333.93(10)	2662.6(11)	1071.2(3)	696.20(8)
*Z*	4	2	4	4	4
*Dc* (Mg m^−3^)	2.000	1.896	1.901	2.385	2.071
μ (mm^−1^)	2.232	1.052	1.197	2.081	1.877
*F* (000)	680	195	1560	752	436
*θ* (°)	3.66 to 26.37	4.01 to 29.46	3.41 to 25.35	3.07 to 25.02	2.74 to 26.37
Data completeness					
Reflections measured	8019	5074	12255	13793	7382
Independent reflections	1146 (*R*_int_ = 0.0331)	1055 (*R*_int_ = 0.0411)	2418 (*R*_int_ = 0.0652)	1878 (*R*_int_ = 0.0512)	1418(*R*_int_ = 0.1497)
Final *R*_1_, *wR*_2_ [*I* > 2σ(*I*)]	0.0393, 0.0867	0.0514, 0.0828	0.0436, 0.0807	0.0432, 0.1128	0.0388, 0.0896
*R*_1_, *wR*_2_ (all data)	0.0568, 0.0944	0.0603, 0.0857	0.087, 0.0925	0.0481, 0.1150	0.0474, 0.0935
Δρ_max/min_ (eǺ^−3^)	0.389, −0.555	0.384, −0.487	0.364, −0.433	0.729, −0.974	0.555, −0.569

*R*_1_ = (*Σ*||*F_o_*| − |*F_c_* || /*Σ* |*F_o_*|). *wR*_2_ = [*Σ* (*w*(*F_o_*^2^ − *F_c_*^2^)^2^)/*Σ* (*w* |*F_o_*^2^|^2^)]^1/2^.

**Table 7:** Highest peaks and deepest holes for structure refinement data for compounds **1**–**5**.

	Δρ_max/_(eǺ^−3^)	Distance/Ǻ	from	Δρ_min/_(eǺ^−3^)	Distance/Ǻ	from
**1**	0.389	0.85	O1W	−0.555	0.70	Zn1
**2**	0.384	1.17	Mn1	−0.487	0.61	Mn1
**3**	0.364	0.52	N24	−0.433	0.64	Fe1
**4**	0.729	0.63	O3	−0.974	1.29	H1A
**5**	0.555	0.90	Mn1	−0.569	0.96	Mn1

## 4. Conclusions

Employing 3-amino-1*H*-1,2,4-triazole-5-carboxylic acid as the organic ligand yielded the first series of five compounds, namely mononuclear [Zn(Hatrc)_2_(H_2_O)] (**1**), [Mn(Hatrc)_2_(H_2_O)_2_]·2H_2_O (**2**)], binuclear [Fe_2_(Hatrc)_4_(OH)_2_]·6H_2_O (**3**), 1D zigzag chain [Cd(Hatrc)_2_(H_2_O)]*_n_* (**4**), and 2D layer [Mn(atrc)(H_2_O)]*_n_*·*n*H_2_O (**5**). All compounds were isolated as single crystals.

In this series, (H)atrc ligands adopt three types of connection fashions: in **1**–**3**, they chelate preferentially with the metal centers via the carboxylic O atom and the 4-position N atom to form a five-membered ring; in **4**, with one more metal center chelated by two carboxylic O atoms, the discrete units can shape a polymeric 1D chain; in **5**, based on the fashion in **4**, two more metal centers are bound through, on the one hand, a similar five-membered ring being formed by the 2-positioned N atom and the second carboxylic O atom and, on the other, the fourth metal being connected to the 1-positioned N atom. The various coordination fashions play, in this respect, a significant role in the structural fabrication of the various compounds. The residual uncoordinated donor sites in the anionic ligands in compounds **1**–**4** open the possibility to use compounds **1**–**4** as precursors of building blocks to further isolate multidimensional frameworks. With the rich existence of donors and acceptors in the present structures, the hydrogen-bonding interactions with the various water molecules incorporated into the materials play key roles in the supramolecular organization leading to the formation of 3D supramolecular architectures.

## Supplementary Materials

Supplementary materials can be accessed at: http://www.mdpi.com/1420-3049/20/07/12341/s1.
