# Supplementary material for: Multidimensional Transition Metal Complexes Based on 3-Amino-1H-1,2,4-triazole-5-carboxylic Acid: From Discrete Mononuclear Complexes to Layered Materials
Source: Molecules. 2015 Jul 7;20(7):12341–63. doi: 10.3390/molecules200712341 (PMC6332153; doi:10.3390/molecules200712341)
Supplement: Supplementary file 1 [file molecules-20-12341-s001.zip › comp_3-CheckCIF.pdf]

checkCIF (full publication check) running

checkCIF/PLATON (full publication check)

Structure factors have been supplied for datablock(s) I

THIS REPORT IS FOR GUIDANCE ONLY. IF USED AS PART OF A REVIEW PROCEDURE FOR PUBLICATION, IT SHOULD NOT REPLACE THE EXPERTISE OF AN EXPERIENCED CRYSTALLOGRAPHIC REFEREE.

No syntax errors found.
Please wait while processing ....

CIF dictionary
Interpreting this report

Structure factor report

Datablock: I

Bond precision:	C-C = 0.0050 A		Wavelength=0.71073
Cell:	a=27.111(6)	b=12.697(3)	c=7.7352(17)
	alpha=90	beta=90	gamma=90
Temperature:	150 K		

	Calculated	Reported
Volume	2662.7(10)	2662.6(11)
Space group	P n n a	P n n a
Hall group	-P 2a 2bc	-P 2a 2bc
Moiety formula	C12 H14 Fe2 N16 O10, 6(H2 O)	C12 H14 Fe2 N16 O10, 6(H2 O)
Sum formula	C12 H26 Fe2 N16 O16	C12 H26 Fe2 N16 O16
Mr	762.19	762.19
Dx,g cm-3	1.901	1.901
Z	4	4
Mu (mm-1)	1.197	1.197
F000	1560.0	1560.0
F000'	1563.51	
h,k,lmax	32,15,9	32,15,9
Nref	2447	2418
Tmin,Tmax	0.972,0.976	0.921,0.965
Tmin'	0.920	

Correction method= # Reported T Limits: Tmin=0.921 Tmax=0.965
AbsCorr = MULTI-SCAN

Data completeness= 0.988 Theta(max)= 25.350

R(reflections)= 0.0436(1587) wR2(reflections)= 0.0925(2418)

S = 1.015 Npar= 247

The following ALERTS were generated. Each ALERT has the format
test-name_ALERT_alert-type_alert-level.
Click on the hyperlinks for more details of the test.

Alert level C

PLAT088_ALERT_3_C Poor Data / Parameter Ratio .... 9.79 Note
PLAT910_ALERT_3_C Missing # of FCF Reflection(s) Below Th(Min) ... 7 Report
PLAT911_ALERT_3_C Missing # FCF Refl Between THmin & STh/L= 0.600 17 Report

Alert level G

PLAT002_ALERT_2_G Number of Distance or Angle Restraints on AtSite 21 Note
PLAT172_ALERT_4_G The CIF-Embedded .res File Contains DFIX Records 16 Report
PLAT720_ALERT_4_G Number of Unusual/Non-Standard Labels .... 6 Note
PLAT790_ALERT_4_G Centre of Gravity not Within Unit Cell: Resd. # 4 Note
H2 O
PLAT860_ALERT_3_G Number of Least-Squares Restraints .... 16 Note
PLAT912_ALERT_4_G Missing # of FCF Reflections Above STh/L= 0.600 5 Note

0 **ALERT level A** = Most likely a serious problem - resolve or explain
0 **ALERT level B** = A potentially serious problem, consider carefully

3 **ALERT level C** = Check. Ensure it is not caused by an omission or oversight
6 **ALERT level G** = General information/check it is not something unexpected

0 ALERT type 1 CIF construction/syntax error, inconsistent or missing data
1 ALERT type 2 Indicator that the structure model may be wrong or deficient
4 ALERT type 3 Indicator that the structure quality may be low
4 ALERT type 4 Improvement, methodology, query or suggestion
0 ALERT type 5 Informative message, check

checkCIF publication errors

Alert level A

PUBL004_ALERT_1_A The contact author's name and address are missing,
_publ_contact_author_name and _publ_contact_author_address.
PUBL005_ALERT_1_A _publ_contact_author_email, _publ_contact_author_fax and
_publ_contact_author_phone are all missing.
At least one of these should be present.
PUBL006_ALERT_1_A _publ_requested_journal is missing
e.g. 'Acta Crystallographica Section C'
PUBL008_ALERT_1_A _publ_section_title is missing. Title of paper.
PUBL009_ALERT_1_A _publ_author_name is missing. List of author(s) name(s).
PUBL010_ALERT_1_A _publ_author_address is missing. Author(s) address(es).
PUBL012_ALERT_1_A _publ_section_abstract is missing.
Abstract of paper in English.

Alert level G

PUBL017_ALERT_1_G The _publ_section_references section is missing or
empty.

7 **ALERT level A** = Data missing that is essential or data in wrong format
1 **ALERT level G** = General alerts. Data that may be required is missing

Publication of your CIF

You should attempt to resolve as many as possible of the alerts in all categories. Often the minor alerts point to easily fixed oversights, errors and omissions in your CIF or refinement strategy, so attention to these fine details can be worthwhile. In order to resolve some of the more serious problems it may be necessary to carry out additional measurements or structure refinements. However, the nature of your study may justify the reported deviations from journal submission requirements and the more serious of these should be commented upon in the discussion or experimental section of a paper or in the "special_details" fields of the CIF. *checkCIF* was carefully designed to identify outliers and unusual parameters, but every test has its limitations and alerts that are not important in a particular case may appear. Conversely, the absence of alerts does not guarantee there are no aspects of the results needing attention. It is up to the individual to critically assess their own results and, if necessary, seek expert advice.

If level A alerts remain, which you believe to be justified deviations, and you intend to submit this CIF for publication in a journal, you should additionally insert an explanation in your CIF using the Validation Reply Form (VRF) below. This will allow your explanation to be considered as part of the review process.

Validation response form

Please find below a validation response form (VRF) that can be filled in and pasted into your CIF.

```
# start Validation Reply Form
_vrf_PUBL004_GLOBAL
;
PROBLEM: The contact author's name and address are missing,
RESPONSE: ...
;
_vrf_PUBL005_GLOBAL
;
PROBLEM: _publ_contact_author_email, _publ_contact_author_fax and
RESPONSE: ...
;
_vrf_PUBL006_GLOBAL
;
PROBLEM: _publ_requested_journal is missing
RESPONSE: ...
;
_vrf_PUBL008_GLOBAL
;
PROBLEM: _publ_section_title is missing. Title of paper.
RESPONSE: ...
```

;
_vrf_PUBL009_GLOBAL
;
PROBLEM: _publ_author_name is missing. List of author(s) name(s).
RESPONSE: ...
;
_vrf_PUBL010_GLOBAL
;
PROBLEM: _publ_author_address is missing. Author(s) address(es).
RESPONSE: ...
;
_vrf_PUBL012_GLOBAL
;
PROBLEM: _publ_section_abstract is missing.
RESPONSE: ...
;
end Validation Reply Form

If you wish to submit your CIF for publication in Acta Crystallographica Section C or E, you should upload your CIF via the web. If your CIF is to form part of a submission to another IUCr journal, you will be asked, either during electronic submission or by the Co-editor handling your paper, to upload your CIF via our web site.

PLATON version of 21/06/2015; check.def file version of 21/06/2015

Datablock I - ellipsoid plot

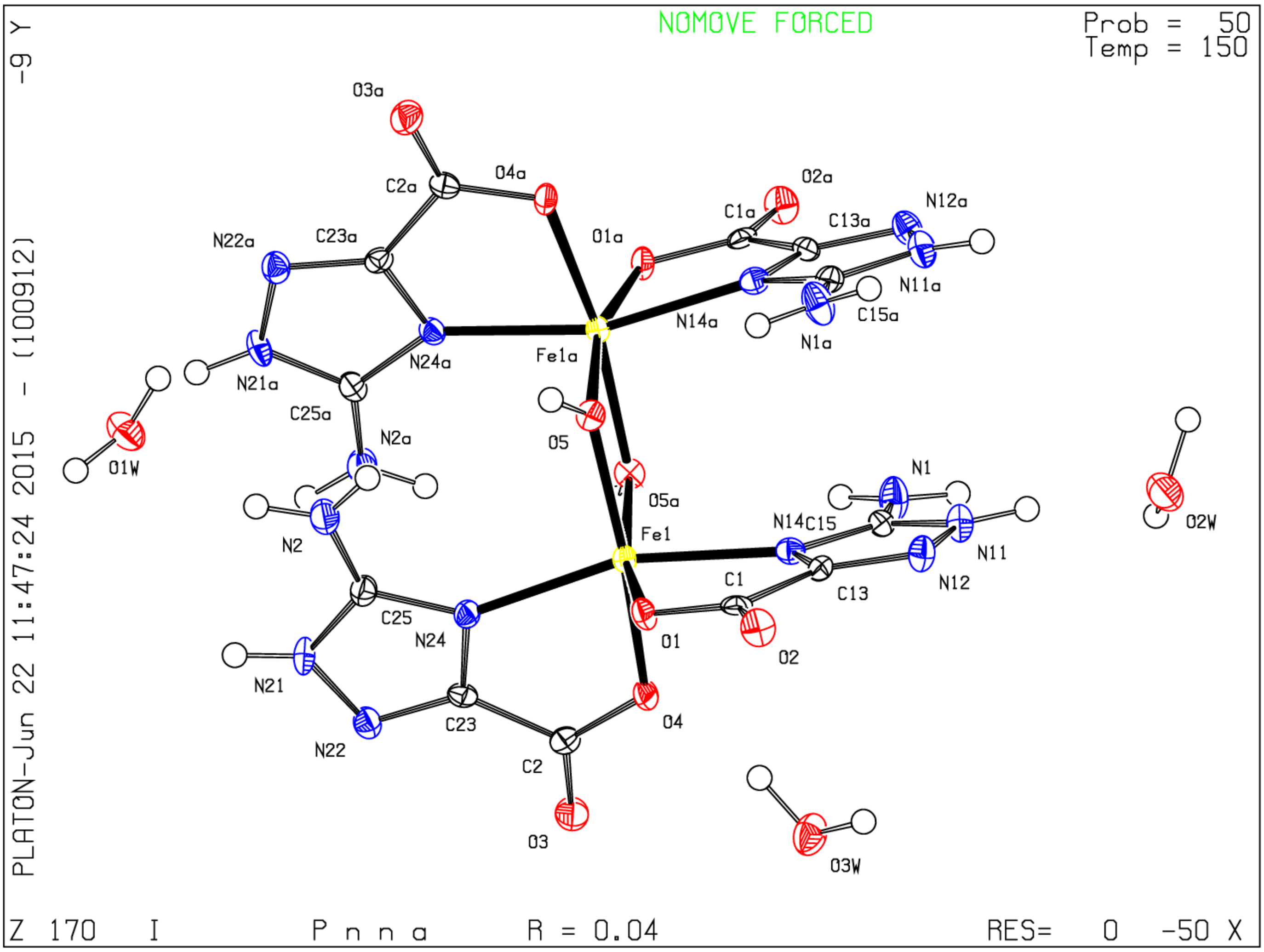

Download CIF editor (publCIF) from the IUCr
Download CIF editor (enCIFer) from the CCDC
Test a new CIF entry
