# Supplementary material for: Multidimensional Transition Metal Complexes Based on 3-Amino-1H-1,2,4-triazole-5-carboxylic Acid: From Discrete Mononuclear Complexes to Layered Materials
Source: Molecules. 2015 Jul 7;20(7):12341–63. doi: 10.3390/molecules200712341 (PMC6332153; doi:10.3390/molecules200712341)
Supplement: Supplementary file 1 [file molecules-20-12341-s001.zip › comp_4-CheckCIF.pdf]

checkCIF (full publication check) running

checkCIF/PLATON (full publication check)

Structure factors have been supplied for datablock(s) I

THIS REPORT IS FOR GUIDANCE ONLY. IF USED AS PART OF A REVIEW PROCEDURE FOR PUBLICATION, IT SHOULD NOT REPLACE THE EXPERTISE OF AN EXPERIENCED CRYSTALLOGRAPHIC REFEREE.

No syntax errors found.
Please wait while processing ....

CIF dictionary
Interpreting this report

Structure factor report

Datablock: I

```
Bond precision:       C-C = 0.0090 A                Wavelength=0.71073
Cell:        a=8.9893(13)      b=8.9785(13)      c=13.9642(16)
             alpha=90          beta=108.117(5)   gamma=90
Temperature: 150 K
                     Calculated                          Reported
Volume               1071.2(3)                           1071.2(3)
Space group          P 21/n                              P 21/n
Hall group           -P 2yn                              -P 2yn
Moiety formula       C6 H8 Cd N8 O5                      C6 H8 Cd N8 O5
Sum formula          C6 H8 Cd N8 O5                      C6 H8 Cd N8 O5
Mr                   384.61                              384.60
Dx,g cm-3            2.385                               2.385
Z                    4                                   4
Mu (mm-1)            2.081                               2.081
F000                 752.0                               752.0
F000'                749.06
h,k,lmax             10,10,16                            10,10,16
Nref                 1886                                1878
Tmin,Tmax            0.905,0.939                         0.905,0.939
Tmin'                0.864
Correction method= # Reported T Limits: Tmin=0.905 Tmax=0.939
AbsCorr = MULTI-SCAN
Data completeness= 0.996           Theta(max)= 25.023
R(reflections)= 0.0432( 1720)         wR2(reflections)= 0.1150( 1878)
S = 1.137                Npar= 205
```

The following ALERTS were generated. Each ALERT has the format
test-name_ALERT_alert-type_alert-level.
Click on the hyperlinks for more details of the test.

Alert level C

PLAT088_ALERT_3_C Poor Data / Parameter Ratio .... 9.16 Note
PLAT213_ALERT_2_C Atom O2 has ADP max/min Ratio ..... 3.2 oblate
PLAT342_ALERT_3_C Low Bond Precision on C-C Bonds .... 0.0090 Ang.
PLAT790_ALERT_4_C Centre of Gravity not Within Unit Cell: Resd. # 1 Note
C6 H8 Cd N8 O5
PLAT906_ALERT_3_C Large K value in the Analysis of Variance ...... 3.147 Check
PLAT911_ALERT_3_C Missing # FCF Refl Between THmin & STh/L= 0.595 6 Report

Alert level G

PLAT002_ALERT_2_G Number of Distance or Angle Restraints on AtSite 13 Note
PLAT003_ALERT_2_G Number of Uiso or Uij Restrained non-H Atoms ... 1 Report
PLAT004_ALERT_5_G Polymeric Structure Found with Maximum Dimension 1 Info
PLAT066_ALERT_1_G Predicted and Reported Tmin&Tmax Range Identical ? Check
PLAT083_ALERT_2_G SHELXL Second Parameter in WGHT Unusually Large. 11.82 Why ?
PLAT172_ALERT_4_G The CIF-Embedded .res File Contains DFIX Records 5 Report

PLAT720_ALERT_4_G Number of Unusual/Non-Standard Labels .... 2 Note
PLAT860_ALERT_3_G Number of Least-Squares Restraints .... 15 Note
PLAT909_ALERT_3_G Percentage of Observed Data at Theta(Max) still 73 %
PLAT910_ALERT_3_G Missing # of FCF Reflection(s) Below Th(Min) ... 2 Report

0 **ALERT level A** = Most likely a serious problem - resolve or explain
0 **ALERT level B** = A potentially serious problem, consider carefully
6 **ALERT level C** = Check. Ensure it is not caused by an omission or oversight
10 **ALERT level G** = General information/check it is not something unexpected

1 ALERT type 1 CIF construction/syntax error, inconsistent or missing data
4 ALERT type 2 Indicator that the structure model may be wrong or deficient
7 ALERT type 3 Indicator that the structure quality may be low
3 ALERT type 4 Improvement, methodology, query or suggestion
1 ALERT type 5 Informative message, check

checkCIF publication errors

Alert level A

PUBL004_ALERT_1_A The contact author's name and address are missing,
_publ_contact_author_name and _publ_contact_author_address.
PUBL005_ALERT_1_A _publ_contact_author_email, _publ_contact_author_fax and
_publ_contact_author_phone are all missing.
At least one of these should be present.
PUBL006_ALERT_1_A _publ_requested_journal is missing
e.g. 'Acta Crystallographica Section C'
PUBL008_ALERT_1_A _publ_section_title is missing. Title of paper.
PUBL009_ALERT_1_A _publ_author_name is missing. List of author(s) name(s).
PUBL010_ALERT_1_A _publ_author_address is missing. Author(s) address(es).
PUBL012_ALERT_1_A _publ_section_abstract is missing.
Abstract of paper in English.

Alert level G

PUBL017_ALERT_1_G The _publ_section_references section is missing or
empty.

7 **ALERT level A** = Data missing that is essential or data in wrong format
1 **ALERT level G** = General alerts. Data that may be required is missing

Publication of your CIF

You should attempt to resolve as many as possible of the alerts in all categories. Often the minor alerts point to easily fixed oversights, errors and omissions in your CIF or refinement strategy, so attention to these fine details can be worthwhile. In order to resolve some of the more serious problems it may be necessary to carry out additional measurements or structure refinements. However, the nature of your study may justify the reported deviations from journal submission requirements and the more serious of these should be commented upon in the discussion or experimental section of a paper or in the "special_details" fields of the CIF. *checkCIF* was carefully designed to identify outliers and unusual parameters, but every test has its limitations and alerts that are not important in a particular case may appear. Conversely, the absence of alerts does not guarantee there are no aspects of the results needing attention. It is up to the individual to critically assess their own results and, if necessary, seek expert advice.

If level A alerts remain, which you believe to be justified deviations, and you intend to submit this CIF for publication in a journal, you should additionally insert an explanation in your CIF using the Validation Reply Form (VRF) below. This will allow your explanation to be considered as part of the review process.

Validation response form

Please find below a validation response form (VRF) that can be filled in and pasted into your CIF.

```
# start Validation Reply Form
_vrf_PUBL004_GLOBAL
;
PROBLEM: The contact author's name and address are missing,
RESPONSE: ...
;
_vrf_PUBL005_GLOBAL
;
PROBLEM: _publ_contact_author_email, _publ_contact_author_fax and
RESPONSE: ...
;
_vrf_PUBL006_GLOBAL
;
```

PROBLEM: _publ_requested_journal is missing
RESPONSE: ...
;
_vrf_PUBL008_GLOBAL
;
PROBLEM: _publ_section_title is missing. Title of paper.
RESPONSE: ...
;
_vrf_PUBL009_GLOBAL
;
PROBLEM: _publ_author_name is missing. List of author(s) name(s).
RESPONSE: ...
;
_vrf_PUBL010_GLOBAL
;
PROBLEM: _publ_author_address is missing. Author(s) address(es).
RESPONSE: ...
;
_vrf_PUBL012_GLOBAL
;
PROBLEM: _publ_section_abstract is missing.
RESPONSE: ...
;
end Validation Reply Form

If you wish to submit your CIF for publication in Acta Crystallographica Section C or E, you should upload your CIF via the web. If your CIF is to form part of a submission to another IUCr journal, you will be asked, either during electronic submission or by the Co-editor handling your paper, to upload your CIF via our web site.

PLATON version of 21/06/2015; check.def file version of 21/06/2015

Datablock I - ellipsoid plot

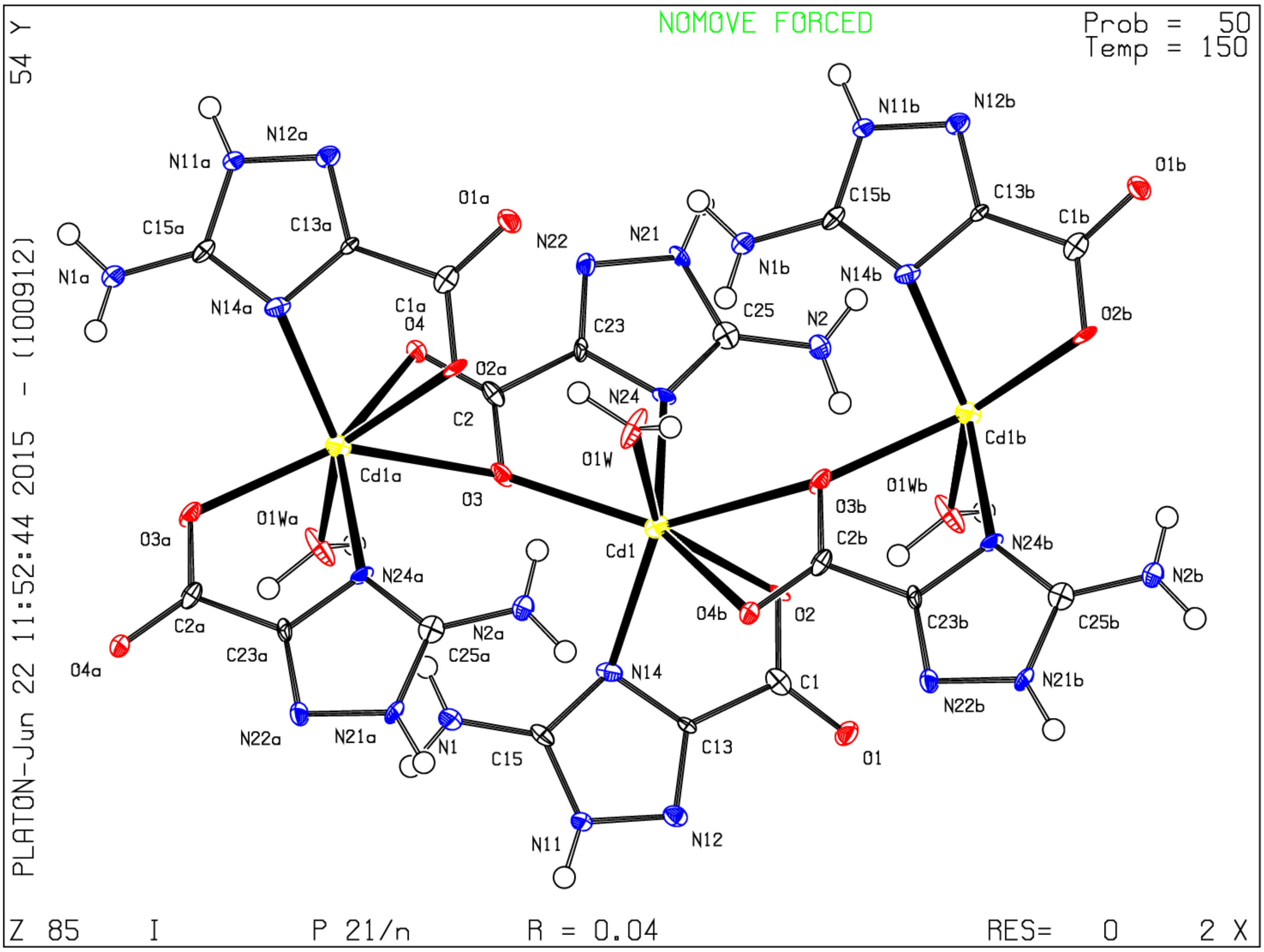

Download CIF editor (publCIF) from the IUCr

Download CIF editor (enCIFer) from the CCDC
Test a new CIF entry
